# Coating of Magnetite Nanoparticles with Fucoidan to Enhance Magnetic Hyperthermia Efficiency

**DOI:** 10.3390/nano11112939

**Published:** 2021-11-02

**Authors:** Joana Gonçalves, Cláudia Nunes, Liliana Ferreira, Maria Margarida Cruz, Helena Oliveira, Verónica Bastos, Álvaro Mayoral, Qing Zhang, Paula Ferreira

**Affiliations:** 1 CICECO-Aveiro Institute of Materials, Department of Materials and Ceramic Engineering, University of Aveiro, 3810-193 Aveiro, Portugal; joanadfgoncalves@ua.pt; 2 Physics Department, University of Coimbra, 3004-516 Coimbra, Portugal; lmferreira@fc.ul.pt; 3 Biosystems and Integrative Sciences Institute (BioISI), Faculdade de Ciências, Universidade de Lisboa, 1749-016 Lisboa, Portugal; mmcruz@fc.ul.pt; 4 CESAM, Department of Biology, University of Aveiro, 3810-193 Aveiro, Portugal; holiveira@ua.pt (H.O.); veronicabastos@ua.pt (V.B.); 5 Instituto de Nanociencia y Materiales de Aragon (INMA), Spanish National Research Council (CSIC), University of Zaragoza 12, Calle de Pedro Cerbuna, 50009 Zaragoza, Spain; amayoral@unizar.es; 6 Advanced Microscopy Laboratory (LMA), Universidad de Zaragoza, 50018 Zaragoza, Spain; 7 Center for High-Resolution Electron Microscopy (CћEM), School of Physical Science and Technology (SPST), ShanghaiTech University, 393 Middle Huaxia Road, Pudong, Shanghai 201210, China; zhangqing1@shanghaitech.edu.cn

**Keywords:** magnetic nanoparticles, magnetite, fucoidan, magnetic hyperthermia therapy, biocompatibility

## Abstract

Magnetic nanoparticles (NP), such as magnetite, have been the subject of research for application in the biomedical field, especially in Magnetic Hyperthermia Therapy (MHT), a promising technique for cancer therapy. NP are often coated with different compounds such as natural or synthetic polymers to protect them from oxidation and enhance their colloidal electrostatic stability while maintaining their thermal efficiency. In this work, the synthesis and characterization of magnetite nanoparticles coated with fucoidan, a biopolymer with recognized biocompatibility and antitumoral activity, is reported. The potential application of NP in MHT was evaluated through the assessment of Specific Loss Power (SLP) under an electromagnetic field amplitude of 14.7 kA m^−1^ and at 276 kHz. For fucoidan-coated NP, it was obtained SLP values of 100 and 156 W/g, corresponding to an Intrinsic Loss Power (ILP) of 1.7 and 2.6 nHm^2^kg^−1^, respectively. These values are, in general, higher than the ones reported in the literature for non-coated magnetite NP or coated with other polymers. Furthermore, in vitro assays showed that fucoidan and fucoidan-coated NP are biocompatible. The particle size (between ca. 6 to 12 nm), heating efficiency, and biocompatibility of fucoidan-coated magnetite NP meet the required criteria for MHT application.

## 1. Introduction

Regardless of efforts to find new therapies and the considerable progress in medical research, cancer is still one of the biggest causes of death in the world [1,2] with an estimation of 18.1 million new cancer cases and 9.6 million deaths in 2018 [3]. Furthermore, an increase to 27.5 million of new cancer cases by 2040 is expected [4]. Conventional treatments, such as chemotherapy (CT), radiotherapy (RT), and surgery, originate side effects (hair loss, bleeding, edema, and fatigue) [5,6], and as such it is extremely urgent that to develop new treatments to overcome these drawbacks with similar or improved efficiency against cancer [1,2]. 

Hyperthermia appears as an adjuvant alternative in the treatment of cancer [7]. The use of hyperthermia arises from the existence of a distinct response and tolerance to heat between healthy and tumor cells. In normal tissues, the heat induces a fast response, which includes an increase of blood flow, accompanied by vessel dilatation and an increase of vascular wall permeability, while in the tumor tissues, the vascular network is less efficient, namely in exchanging heat, and, consequently, it is more likely to be damaged when treated by hyperthermia therapy. The cytotoxic effect is mainly associated with the fact that an increase in temperature leads to a denaturation of cytoplasmic proteins, influencing the cell growth and differentiation which in turn can induce apoptosis [5,8,9].

The currently available regional or local hyperthermia modalities have the difficulty of heating the tumor homogeneously, since a temperature gradient is generated between the tumor surface and its inner core, reducing the treatment’s efficiency [8]. Additionally, conventional hyperthermia may also affect healthy cells due to non-selective heating [1,7]. Magnetic nanoparticle-mediated hyperthermia, also known as Magnetic Hyperthermia Therapy (MHT), has the potential to overcome this limitation using superparamagnetic nanoparticles (NP) such as magnetite (Fe_3_O_4_) and maghemite (γ-Fe_2_O_3_) to produce homogenous therapeutic heating (in the range of 41 to 46 °C), confined to the tumor tissue [2,7,10], reducing the negative side effect common in conventional cancer therapies such as CT and RT [9]. For Fe_3_O_4_ and γ-Fe_2_O_3_, superparamagnetic behavior is typical of NP with sizes below 30 nm and is highly required to avoid embolization [2].

MHT has already shown its therapeutic efficacy in clinical trials, mostly as a combinatorial approach with other conventional therapies such as radiotherapy or chemotherapy [10,11,12]. However, an inevitable issue associated with the use of particles in this size range (less than 20 nm) [13] is their intrinsic instability [14,15], since NP tend to aggregate over long periods of time due to their large surface area-to-volume ratio [5,15,16] and also due to the magnetic dipolar attraction between the NP [17]. In addition, iron oxide nanoparticles are easily oxidized in air [14]. Both these issues undermine their heating efficiency [15], characterized by the Specific Loss Power (SLP). For MHT applications, NP with high SLP are needed to achieve the clinical desired temperature without requiring a high NP dose to be administered to the patient [18].

Surface coating is one of the strategies used to overcome these drawbacks, allowing protection from oxidation, improving biocompatibility, and enhancing their colloidal stability [13,19].

NP are often coated with different materials including organic (e.g., dextran, starch, and chitosan) and inorganic coatings (e.g., gold, silver, carbon, and silica) [13,20] during (in situ) or after their synthesis (post-synthesis) [13,21].

Furthermore, the search for new sources of raw compounds to be used in the biomedical field has led to the use of sulfated polysaccharides from marine algae, namely fucoidan due to its biocompatibility [22] and antitumor properties [23,24,25,26,27]. The use of fucoidan as coating agent prevented NP agglomeration, increasing NP magnetization [28]. Fucoidan has been revealed to possess anticancer activity playing a role in the reduction of the tumor size [29]. A synergetic effect with other cancer therapies such as photothermal therapy has been also demonstrated [30]. The use of fucoidan to control drug delivery from the pores of a mesoporous silica shell around a magnetite core has also been described. It was verified that fucoidan was responsive to pH and temperature, allowing the release of the drug. According to the authors, the particles could be used for magnetic hyperthermia [31].

It is known that tumors can destabilize our immune system by stimulation of a variety of immune suppression mechanisms in the tumor and microenvironment. Furthermore, cancer therapy-based CT may also have side effects related to drugs that induce immunosuppression. Immunomodulators can be used as immunostimulants by stimulating immune cells to enhance antitumor effects [32]. Fucoidan has been reported to present immunostimulatory activity [33,34,35] and to suppress cancer growth by increasing the body’s immunity [35,36]. The conjugation of fucoidan-dextran with magnetic particles to achieve an immunosuppressive tumor microenvironment has also been reported [37]. The magnetic particles allow for conducting the fucoidan to the tumor, for localized immunosuppressive activity, avoiding, in this way, off-target effects.

A few reports can be found in literate of the use of fucoidan associated to NP as coating agent, aiming to improve the NP stabilization, or taking advantage of their anticancer biological property. For instance, Silva et al. [28] used fucoidan to coat magnetite NP to study its influence on NP stabilization and magnetic properties. The same group also investigated the anticancer property of fucoidan [29]. However, only the resulting magnetic properties and the anticancer activity of fucoidan were investigated. The authors did not address the application of these particles on MHT. The biological activity of fucoidan makes it an agent with great potential in the treatment of cancer. The coating of magnetite NP with fucoidan is an opportunity to achieve multifunctional nanoparticles, combining the thermal hyperthermia efficiency of magnetite with the intrinsic anticancer property of fucoidan. In this work, it is described the development of magnetite NP coated with fucoidan for cancer therapy. These NP were synthesized by co-precipitation which is a relatively easy way to produce superparamagnetic magnetite NP without using hazardous reagents and offering a low-temperature alternative to the other conventional methods [13]. The coating was performed using two approaches: (i) after their synthesis (post-synthesis coating), and (ii) simultaneously with the synthesis (in situ coating). To evaluate the NP potential for MHT, the heating efficiency under an alternating magnetic field was determined and assays were performed to assess cell viability in contact with fucoidan, pristine NP and fucoidan-coated magnetite NP.

## 2. Materials and Methods

*Materials*: Ferrous (II) sulfate heptahydrate (FeSO_4_·7H_2_O, purity 99.5%) and iron (III) chloride (FeCl_3_, purity 97%) were purchased from Merck. Ammonium hydroxide (NH_4_OH, 25 vol%) was purchased from Sigma Aldrich. Fucoidan powder was obtained from Shandong Jiejing group (batcher no. 131023). All the reagents were used without any further purification.

*Synthesis of pristine Nanoparticles:* The protocol employed in the magnetite nanoparticles synthesis was reported in the literature through the addition of a precipitating agent to a stoichiometric aqueous solution of Fe^2+^ and Fe^3+^ in a molar ratio of 1:2 under an oxygen-free environment [38]. An aqueous solution was prepared by dissolving 0.0038 mol of FeSO_4_·7H_2_O and 0.0076 mol of FeCl_3_ at room temperature under a nitrogen atmosphere to avoid oxidation of the ferrous ions. Thereafter, 3.33 mL of NH_4_OH was added and magnetic stirring was maintained for 15 min, producing the black suspension of Fe_3_O_4_ NP. The particles were aged at room temperature for 1 h and then magnetically separated and washed several times with distilled water to remove the excess of NH_3_ until pH 7 was attained. The NP were kept in this solution until they were used. 

*Post-synthesis coating of Fe_3_O_4_ nanoparticles with fucoidan:* The post-synthesis coating was accomplished by the adsorption method based on a study reported by Silva et al. [28]. A 2 mg mL^−1^ fucoidan solution was filtered using a syringe with a disposable filter (hydrophilized polytetrafluoroethylene membrane with a pore size of 0.2 µm) and added to the previously obtained Fe_3_O_4_ NP. This suspension was maintained at room temperature under magnetic stirring for 16 h. Then, the NP were washed with distilled water and kept in the solution until they were used.

*Simultaneous synthesis of Fe_3_O_4_ nanoparticles and coating with fucoidan:* To produce an in situ coating of magnetite NP, an adaptation of the methodology described by Yew et al. [39] was performed. Stoichiometric quantities of the irons Fe^2+^ (0.0038 mol of FeSO_4_·7H_2_O) and Fe^3+^ (0.0076 mol of FeCl_3_) were added to the solution of fucoidan (2 mg mL^−1^) under a nitrogen atmosphere at room temperature under magnetic stirring. Afterwards, 3.33 mL of NH_4_OH was added and magnetic stirring was maintained for 1 h to complete the reaction. Then, the supernatant was removed from the solution using a permanent magnet and the NP were washed with distilled water until pH 7 was reached.

*Analysis of chemical composition of fucoidan:* The alditol acetates method was performed, following the procedure already reported [40], to determine the content in monosaccharides of fucoidan. This was achieved through the polysaccharide hydrolysis to monosaccharides followed by a reduction and acetylation into alditol acetates and analysis by gas chromatography with a flame ionization detector (GC-FID). The sugars were identified by retention time in comparison with standards. Uronic acids were determined by the m-phenylphenol colorimetric method and following the procedure described by Selvendran el al. [41]. The content of uronic acids was calculated by comparison with a calibration curve of galacturonic acid. To complement the fucoidan composition characterization, in terms of sulfate content, elemental analyses was also carried out and accomplished by combustion analysis. For this, Truspec Micro 630-200-200 equipment was used with a combustion furnace temperature and afterburner temperature of 1075 °C and 850 °C, respectively.

*Nanoparticles characterization:* In order to evaluate the functionalization of the pristine NP with fucoidan Fourier transform infrared (FTIR) spectra was performed over the range of 4000–500 cm^−1^ with Perkin Elmer Spectrum BX with a resolution of 4 cm^−1^ and 64 scans per sample.

The analysis of C (carbon), H (hydrogen), N (nitrogen), and S (sulfur) content was also carried out using Truspec Micro 630-200-200 equipment with the same conditions mentioned above for fucoidan analysis.

To all NP synthetized, pristine and fucoidan-coated, X-ray diffraction (XRD) analysis was performed to evaluate the crystal structure and crystallite size of NP. The X-ray diffractograms were obtained in a diffractometer (Philips Analytical PW 3050/60 X’ Pert PRO (*θ*/2*θ*)) with Cu-Kα radiation (λ = 1.54060 Å). The diffractograms were obtained by scanning in the 2*θ* range from 20 to 70° with a step of 0.02° at room temperature. Through the broadening of the X-ray diffraction peaks, considering the instrumental correction and assuming a spherical shape, the crystallite size was determined using the Debye–Scherrer Equation [42].

Transmission Electron Microscopy (TEM) and high-resolution TEM (HRTEM) analysis were performed to evaluate the NP morphology, shape, and size. TEM images were obtained using the equipment JEOL 2200FS with an acceleration potential of 200 kV. Spherical aberration corrected (C_s_-corrected) HR(scanning, S)TEM HAADF & ABF (high angle annular dark field & annular bright field) images were carried out in a double aberration-corrected equipment JEOL JEM-ARM300F GRAND ARM with cold FEG which was operated at 300 kV. The column was equipped with JEOL spherical aberration correctors which were tuned before every observation assuring a point resolution of 0.7 Å at 300 kV. The microscope was also equipped with a JEOL EDX spectrometer and a Gatan Quantum Energy Filter. The ImageJ^®^ program [43] was used to estimate the mean size of the NP and also the particle-size distributions (a minimum of 100 particles was used for each sample). The histograms were fitted assuming a Log-Normal distribution of the NP diameters and the particle size distribution of the samples was determined statistically.

Brunauer-Emmett-Teller (BET) was used to determine the specific surface area (SSA) of all the synthetized NP [44]. The equipment used was a Micromeritics ^®^—Gemini 2380 V2.00. The samples were degassed overnight at 150 °C to remove physically adsorbed water, which would interfere with the surface area results. Before analysis, the samples were cooled down to −196 °C using liquid nitrogen. The specific surface area of the materials was determined from nitrogen gas adsorption–desorption.

The colloidal stability was accessed through measurements of the zeta potential in MilliQ water and following the procedure used by Bini et al. [45]. Water was used since it is a fluid that can be administrated to the human body. The zeta potential was determined as a function of pH at room temperature using a Zetasizer Nano ZS from Malvern Instruments.

Using a SQUID magnetometer (QD-MPMS), magnetization measurements on the frozen emulsions of nanoparticles were carried out as a function of temperature between 10 and 250 K, after cooling from room temperature in zero magnetic field (zero field cooled—ZFC) and after cooling under the measurement field (field cooled—FC). Hysteresis cycles were obtained at 250 K for applied magnetic fields up to 2 mT. Minor cycles up to 16 kA m^−1^ were also acquired.

The assessment of the nanoparticles’ Specific Loss Power (SLP) under an electromagnetic field was carried out using an Easy Heat 0224 device (Ambrell) based system, using a two-turn Helmholtz solenoid of around 6 cm diameter and working at 276 kHz frequency with an AC field amplitude of 14.7 kA m^−1^. Heat losses and the influence of coil heating were reduced by a layer of thermal insulation placed between the coils and the sample.

In order to avoid harmful heating in the patients related to the electromagnetic radiation exposure, it was reported that for a safe MHT, the H_0_*f* factor should not exceed a limit equal to 5 × 10^9^ Am^−1^ s^−1^ known as “Brezovich limit” [20]. Accordingly, in this work the SLP measurements were carried out below threshold (4.057 × 10^9^ Am^−1^ s^−1^). The experimental setup was non-adiabatic and the protocol used to determine the SLP of the samples was previously reported [2].

*In vitro cytotoxicity assays:* Highly pigmented human melanoma MNT-1 cell line was kindly provided by Dr. Manuela Gaspar (iMed. ULisboa, Portugal). Different concentrations of fucoidan (0.25, 0.5, 1, and 2 mg mL^−1^), pristine NP, and fucoidan-coated NP (0.0125, 0.025, 0.05, 0.1, and 0.2 mg mL^−1^) were added to the cell line to study the dose-dependent effect. Cells were incubated at 37 °C for 24 and 48 h to evaluate the time-dependent effect. Cell viability upon exposure to fucoidan, pristine NP, and fucoidan-coated NP was determined by the colorimetric 3-(4,5dimethyl-2-thiazolyl)-2,5-diphenyl tetrazolium bromide assay (MTT) [46]. The absorbance was measured at 570 nm using a Synergy HT Multi-mode Microplate Reader (BioTek Instruments). According to the literature, NP may interfere in the optical measurement and/or a side reaction with the assay components may happen [47,48,49]. Thus, to avoid or reduce this, an additional step was introduced which included washing with PBS befo38re introducing the MTT solution.

Cell viability was calculated as a percentage of control cells (without fucoidan/NP) as: (Abs_sample_ − Abs_DMSO_)/(Abs_control_ − Abs_DMSO_) × 100. Data were expressed as the mean ± standard deviation (SD) of at least three independent experiments with three technical replicates each.

## 3. Results and Discussion

In this work, fucoidan-coated magnetite NP were synthetized and the effect of the fucoidan coating on NP stabilization and on the hyperthermia response was investigated. These NP were synthesized by the standard precipitation method. The fucoidan was coated after their synthesis (post-synthesis coating) or simultaneously with the synthesis (in situ coating). The pristine NP (M), post-synthesis (MF) and in situ coated NP (MF−IS) samples were studied.

As fucoidan presents a complex structure due to the existence of different monosaccharides and their linkages, as well as the distribution of the sulfate groups, the fucoidan used in this study was characterized [50]. The obtained results can be observed in Table 1.

Fucoidan is mainly constituted by fucose (418 µg/mg_fucoidan_), galactose (230 µg/mg_fucoidan_) and mannose (51 µg/mg_fucoidan_). The fucose content is within the range reported for fucoidan (34–44% *w*/*w*), [51] but is a galactose-rich one. The fucoidans extracted from brown algae are heteropolymers with diverse backbones constituted by neutral sugars and/or uronic acid residues [27]. For example, fucoidans from the *Sargassum stenophyllum* and *Sargassum siliquosum* were described to be constituted by fucose and galactose as major components, being described as a galactofucoidan [52,53].

The sulfate groups are considered a key factor on fucoidan bioactivity because of their role in the inhibition of the growth of cancer cells [54]. The number of sulfate groups in the fucoidan contributes to the effectiveness of its anti-angiogenic and antitumor activities [51,55,56]. Therefore, the content of this element has an important influence on fucoidan composition. By the elemental analysis results, the sulfate content was 104.5 µg/mg_fucoidan_ (~11%) which is within the range reported for fucoidan extracted from *Ascophyllum nodosum*, *Sargassum kjellmanianum*, *Sargassum thunbergii*, presenting a percentage of sulfate content between 8 and 25% [26]. In addition, fucoidan has also traces of nitrogen (0.4%) which may be associated with the existence of some compounds containing amino groups (e.g., protein or amino sugars) [57].

### 3.1. Structural and Morphological Analysis of NP

Different characterization techniques were used to evaluate and study NP properties such as size, morphology and stability of the non-coated NP (M) and their post-synthesis coating with fucoidan (MF), and NP coated with fucoidan in situ (MF−IS).

**Figure 1:**
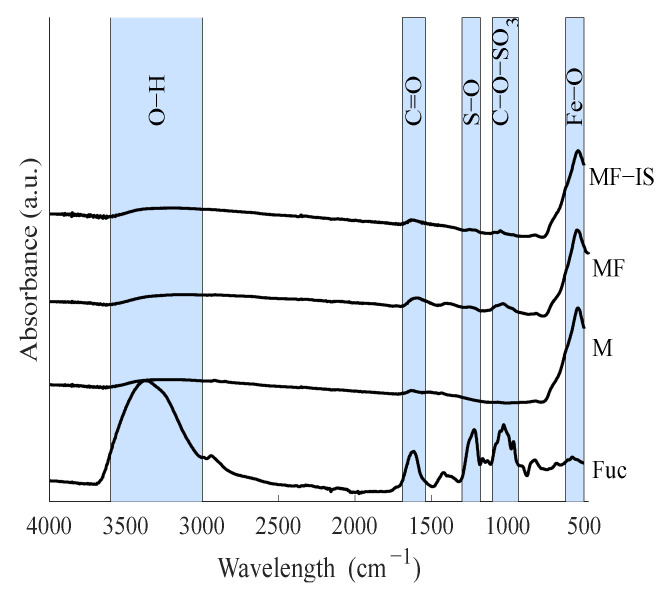
Normalized FTIR spectra of fucoidan (Fuc), magnetite (M), post-synthesis fucoidan-coated magnetite (MF), and in situ fucoidan-coated magnetite (MF−IS) samples.

Fourier-transform infrared spectroscopy (FTIR) spectra of the synthetized samples (M, MF, and MF−IS), as well as the fucoidan (Fuc) for comparison purposes, is depicted in Figure 1.

The magnetite (M) spectrum has a characteristic peak around 570 cm^−1^, which corresponds to the stretching vibrations of metal at the tetrahedral site of Fe-O, confirming the existence of the Fe_3_O_4_ NP core [58]. This peak can be also observed in the spectra of both coated Fe_3_O_4_ NP. The spectra relative to the magnetite coated with fucoidan after synthesis and coated in situ exhibit peaks around 1030 and 1224 cm^−1^. The peak at 1030 cm^−1^ corresponds to symmetric C-O vibration associated with C-O-SO_3_ and the peak at 1224 cm^−1^ is assigned as S=O stretching vibration, which indicates the presence of esterified sulfate. The expression of these peaks comes from the fucoidan [59]. A higher expression of the peak can also be seen around 1628 cm^−1^ (stretching vibration of the carbonyl group in carboxylic acid groups (C=O) in the spectra of the samples with fucoidan (MF, MF−IS) than bare magnetite (M). These results allow the confirmation of the presence of fucoidan on the NP’ surface [59].

The existence of the biopolymer on the NP’ surface is also corroborated by the presence of sulfur determined in the elemental analysis. The post-synthesis fucoidan-coated magnetite sample (MF) showed 11.5% of sulfate content, whereas the in situ coated magnetite sample (MF−IS) had a lower value, 2.2%. This difference agrees with a lower expression exhibition of the fucoidan groups in the MF−IS FTIR spectrum when compared with MF sample (Figure 1). The higher content of fucoidan in the post-synthesis coating may be due to the higher coating time (16 h) than for the NP’ coating in situ which was only 1 h.

**Figure 2:**
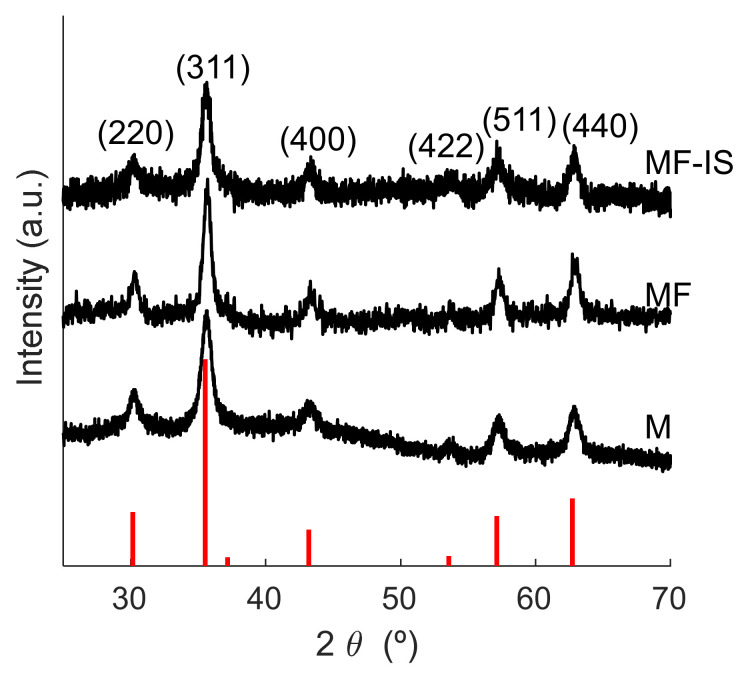
Normalized XRD diffractograms of magnetite (M), post-synthesis fucoidan-coated magnetite (MF), and in situ fucoidan-coated magnetite (MF−IS) samples. The red lines represent the peaks of the Fe_3_O_4_ phase.

X-ray diffraction (XRD) patterns of the synthesized samples are shown in Figure 2. For all samples, the peaks corresponding to the diffraction planes (220), (311), (400), (422), (511), and (440) can be observed and matched with the standard magnetite XRD patterns (S.G. *Fd-*3*m*, JCPDS file No. 04-002-3668). Moreover, the presence of magnetite peaks in the fucoidan-coated samples demonstrates that the coating did not affect the NP core, as already reported [28,29].

**Figure 3:**
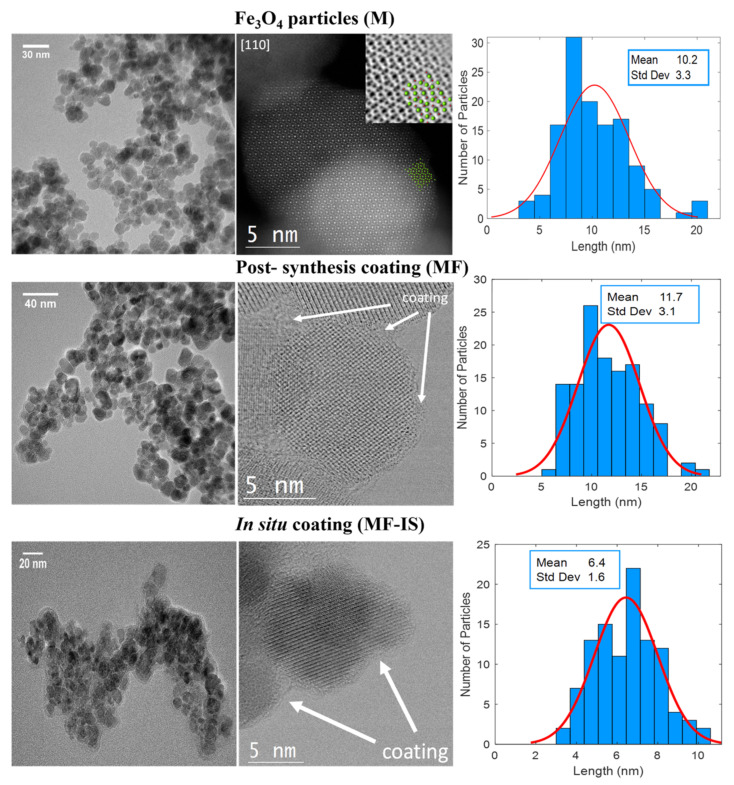
TEM images: low magnification TEM (**left**), Cs-corrected HRSTEM (**center**) and size distributions (**right**) of magnetite (M), post-synthesis fucoidan-coated magnetite (MF), and in situ fucoidan-coated magnetite (MF−IS) samples.

Figure 3 shows the Transmission Electron Microscopy (TEM) images and size distributions of the samples. The pristine magnetite NP are quasi-spherical and present some polydispersity and agglomeration due to the high surface-to-volume ratio [16] and the dipole-dipole magnetic interactions between NP [17,60]. The atomic structure can be directly visualized through C_s_-corrected STEM. As an example, by looking at the non-coated NP, top images, the good crystallinity along the [110] orientation is evidenced. The C_s_-corrected STEM-HAADF micrograph depicts an elongated NP (particle size ≈ 15 nm), where the Fe columns are clearly resolved (see model superimposed, where Fe is represented by green spheres and O in red). In this mode, the contrast is dependent on the atomic number (Z) of the elements and on the number of atoms per column, observing a strong central signal attributed to a Fe column that contains more Fe per unit cell than the surrounding ones that are also formed by Fe. As it is Z dependent, oxygen is not visible in this mode. ABF data was also recorded (see inset in Figure 3) where the contrast is reversed in comparison to the HAADF, allowing the visualization of all atoms (including oxygen) of the structure, for better understanding the model has been also superimposed. Due to the agglomeration of the uncoated NP, the coating in agglomerated zones is more around groups of particles instead of in individualized NP, as can be seen in MF NP. This agglomeration effect on the coating was also already reported in the literature [61]. In this case, a closer look of an individual NP of 12 nm situated on the edge of a group formed by few more was observed along the [001] orientation. Through these images it is not perceptible if the coating provided higher NP dispersion according to their stabilizing effect reported in the literature, where a higher NP dispersion after coating with fucoidan was observed [28,29]. Regarding the in situ coating synthesis with fucoidan (MF−IS), no significant differences were observed except for the reduction in the average particle size suggesting that the fucoidan prevented at some point the initial agglomeration of the NP. Regarding the crystallinity no differences were found in comparison with the other two samples and all crystallized as magnetite with cubic *Fd*$\bar{3}$*m* symmetry. Concerning the coating, it mostly includes groups of particles (clusters) than individual particles.

The mean NP size values, estimated through Debye-Scherrer equation, are presented in Table 2. In addition, the mean particle size obtained through TEM images and specific surface area of all the samples are also displayed. In general, the sizes obtained from Debye-Scherrer equation are in agreement with the results obtained from TEM images. The mean NP size of pristine magnetite (10.2 nm) is in accordance with the reported value for particles prepared by the precipitation method [28,38,62,63,64]. Furthermore, this size is in the proposed range (8 and 20 nm) for application on MHT [2,65,66,67]. Coating the sample M with fucoidan (sample MF) corresponds to a slight increase of their medium size to 11.7 nm. Regarding NP coated in situ (MF−IS), they exhibit a smaller size than the M and MF samples, with an average diameter of 6.4 nm, which corresponds to a higher surface-to-volume ratio, that can explain their greater agglomeration [5].

Furthermore, in situ coated NP have a narrower size dispersion, than NP coated post-synthesis, due to their smaller size [68]. These results are consistent with the Brunauer-Emmett-Teller (BET) measurements since we obtained a specific surface area (total surface area of a material per unit of mass) of 96.3, 91.3, and 111.8 m^2^g^−1^ for M, MF, and MF−IS samples, respectively (Table 2).

**Figure 4:**
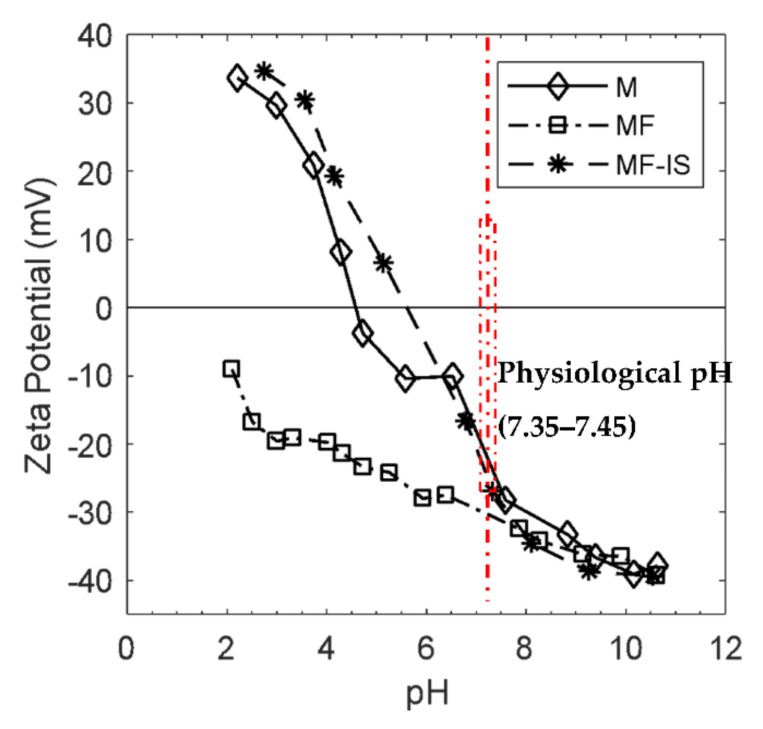
Zeta potential curves as a function of pH of magnetite (M), post-synthesis fucoidan-coated magnetite (MF), and in situ fucoidan-coated magnetite (MF−IS).

Zeta potential curves obtained through the measurement of zeta potential at different pH values are exhibited in Figure 4.

In all the curves for the pH biological range (7.35–7.45) the zeta potential values are all negative which means that the particles’ surface is negatively charged. Observing the curve of the sample M verifies that the isoelectric point is approximately 4.6 which is lower than that reported for synthetic magnetite (~7) [69], which may be due to a possible oxidation of the NP’ surface [69,70]. However, different values have been obtained from different authors (e.g., 3.0–4.0, [71], 4.5 [70], 4.9 [72], 5.0 [73], 5.2 [74], 7.30 [45], 8.0 [47,75]). Based on this value, synthetized NP have a positive charge at pH below 4.7 and negative charge above 4.7, with the predomination of FeOH^+2^ and FeO^−^ groups on magnetite surface respectively. In this work, the adsorption of fucoidan was carried out near pH 7 according to Toi et al. [76]. 

Considering the isoelectric point mentioned above, this pH was not suitable for obtaining the maximum chemisorption of the fucoidan. This condition does not favor the adsorption of the sulfate groups of fucoidan because of the electrostatic repulsion between negative charged magnetite (FeO^−^) and negative charged sulfate groups. However, pH 7 is the required pH to be applied in the biological environment [2].

Regarding the coating of M sample (MF) the NP charge is always negative due to the complexes formed by the NP surface and fucoidan groups and the absence of an isoelectric point can be explained as a consequence of the coating. It is noteworthy that there was an increase of zeta potential value from −19 to −28 mV with the coating producing a higher electrostatic repulsion between the particles which in turn enhance NP stabilization. This can be explained by the presence of fucoidan sulfate groups on the NP surface, since fucoidan is an anionic (negative charge) sulfated polysaccharide [77]. Additionally, negative zeta potential values are in accordance with other reports for NP coated with fucoidan [37,78,79]. 

MF−IS sample have zeta potential values lower than those obtained for NP coated with the post-synthesis method, which may be due to their high surface-to-volume ratio and the tendency to reduce free surface energy. It presented an isoelectric point at 5.5 and −16.7 mV at pH 7. These results confirm the existence of a greater agglomeration of the NP, as already observed in the TEM images. In addition, these results are in accordance with the FTIR and elemental results exhibiting a much lower expression of fucoidan groups in comparison with sample coated post-synthesis. 

For biological applications, stable suspensions at pH around 7.35–7.45 (respective to human blood) are required to avoid NP aggregation and consequently a possible embolization of blood vessels. For this, it is necessary that the zeta potential value (indirect indicator of NP stability) of the NP suspension is usually greater than 25 mV in module [13,38] so that there is a sufficient electrostatic repulsion force to compensate the attraction forces, such as the Van Der Waals forces and magnetic dipole interactions between the NP, avoiding their agglomeration when dispersed into high ionic strength solvents such as biological media. Thus, magnetite post-synthesis coated with fucoidan (MF) exhibits the required stability for biomedical applications. 

### 3.2. Hyperthermia Measurements

To assess the NP potential for MHT, the heating efficiency was evaluated by Specific Loss Power (SLP).

**Figure 5:**
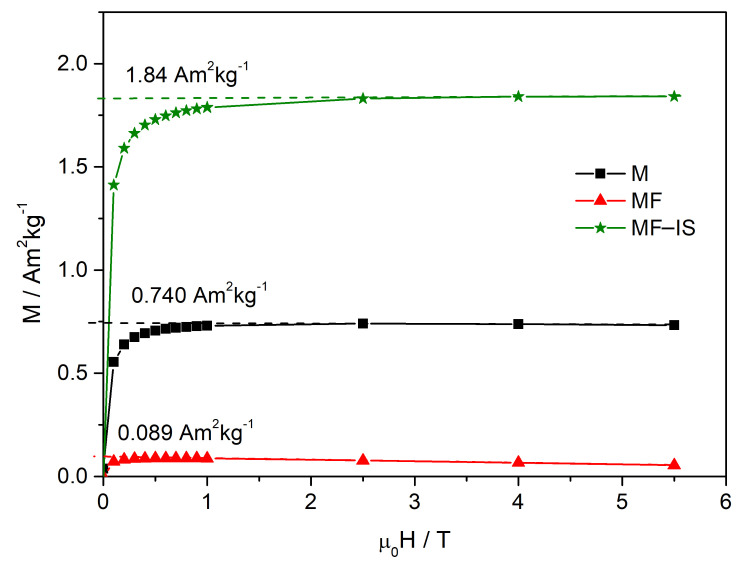
Magnetisation curves M(H) of magnetite (M), post-synthesis fucoidan-coated magnetite (MF), and in situ fucoidan-coated magnetite (MF−IS).

The magnetic hysteresis curves show no coercivity (see Supplementary Materials Figures S1–S3), in agreement with superparamagnetic behavior. Figure 5 shows the magnetization curves (M(H)) of the frozen liquid samples. In this measurements, the spontaneous magnetization values, Ms, correspond to the magnetic NP existing in the measured emulsion volume. Assuming that the saturation magnetization of the bulk magnetite is 92 Am^2^ kg^−1^, the magnetite mass was obtained from the experimental value Ms, and was used to calculate the magnetite concentration in each sample. From the values shown in Figure 5, the magnetite concentrations 8.1 mg mL^−1^, 0.97 mg mL^−1^ and 20 mg mL^−1^, were obtained for emulsions M, MF and MF−IS respectively, indicating that the highest magnetite concentration is obtained for MF−IS, more than twice the one of M, and one order of magnitude above the one of MF.

This concentration was them used to determine the magnetic mass used in the hyperthermia measurements, allowing to normalize the measurements and to obtain the corresponding SLP.

**Figure 6:**
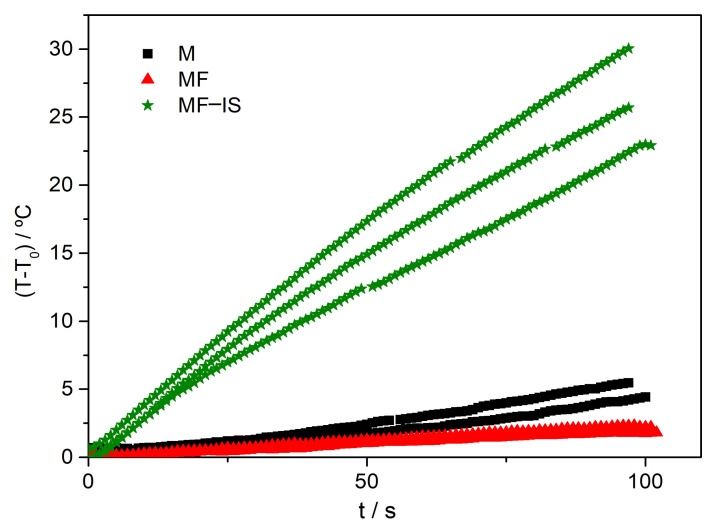
Temperature variation over time (first 100 s) for M, MF and MF−IS NP dispersed in distilled water under the alternating magnetic field.

From the temperature evolution curves illustrated in Figure 6, the mean specific loss power was determined. Three measurements were performed for each sample for time intervals below or equal to 100 s, avoiding major deposition of the NP during measurement. Overall, a gradual increase in temperature with time can be observed. Although a constant NP heat dissipation is expected, a slightly curvature correlated with heat losses can be noted. The results were fitted taking into consideration a constant magnetic heating power released from the NP, assuming linear exchanges (conduction and convection processes) between the environment and the sample and considering a linear variation between the initial and final recorded temperatures of the surrounded environment. The applied fitting expression was [2]:

(1) $$T=T_{0}\;e^{-B\left(t-t_{0}\right)}+\left(T_{0}^{\mathrm{ext}}+\frac{h-\alpha }{B}\right)\left(1-e^{-B\left(t-t_{0}\right)}\right)+\alpha \left(t-t_{0}\right),$$

where *h* and *B* represent, respectively, the magnetic heating power and linear losses coefficient, both divided by the system heat capacity, *T* is the sample temperature at instant *t*, *T*_0_ and $T_{0}^{\mathrm{ext}}$ are the initial sample and environment temperatures at *t*_0_, and α is the linear coefficient for the environment temperature variation.

From this fitting, the parameter *h* was used to determine the specific loss power according to the following expression [2]:

(2) $$SLP_{\mathrm{fit}}=C_{c+w}h\frac{1}{m_{NP}\;}$$

where $C_{c+w}$ is the heat capacity of the container and water, *h* is the magnetic heating power normalized by the system heat capacity and $m_{NP}$ is the mass of the magnetic nanoparticles. Various approaches exist for calculating the SLP from heating/cooling curves. Since the Corrected Slope Method (CSM) [80] is reported to analyze and compensate for the environmental heat losses in non-adiabatic systems, the expression (1) used in this work, deduced for our system, has been previously compared with CSM results for the same samples, showing excellent agreement in the determination of the magnetic heating power.

In the Supplementary Materials are also displayed the temperature dependence of the magnetization (Figure S1), and the hysteresis (Figure S2) and minor hysteresis curve (Figure S3) at 250 K for MF−IS sample. The SLP values obtained for the different samples are summarized in Table 3. 

The thermal performance of magnetic NP increases as a function of frequency (*f*) and field amplitude (H) [1]. Thus, to compare the obtained results with the ones reported in the literature, performed at different experimental conditions of amplitude and frequency, the corresponding intrinsic loss power (ILP) values are also included on Table 3 and were calculated by ILP = SLP/H^2^*f* [4,81]. However, this parameter may be applied to compare the outcomes of heating efficiency only when relatively low field strengths (few kA/m) and frequencies (several hundred kHz) are used [4,82], in which the magnetic susceptibility is assumed to be independent of the magnetic field. Since for clinical magnetic hyperthermia applications it is recommendable to respect the acceptable limits regarding the AMF field and frequency, and NP should be superparamagnetic, this is considered a good approximation [2,81].

The pristine magnetite NP with a medium size of 10.2 nm originated a SLP value of 30 Wg^−1^ corresponding to an ILP of 0.5 nHm^2^kg^−1^. This value is higher than some reported values for pristine NP using the co-precipitation method (Table 4). For instance, Giri et al. [83], synthetized magnetite NP with similar sizes of 10–12 nm and obtained a lower ILP value (0.22 nHm^2^kg^−1^). Likewise, Senturk and co-workers produced magnetite NP with 8.3 ± 1.6 nm and an ILP of 0.23 was obtained [84]. This value is also higher than the value obtained by Shete et al. [85] even for a higher magnetite NP size (21.8 ± 5 nm, 0.42 nHm^2^kg^−1^). More recently, Younis et al. [86] attained 0.22 nHm^2^kg^−1^ for NP with 13 nm ± 1 nm.

Concerning the post-synthesis coating with fucoidan (sample MF, mean size of 11.7 nm ± 3.1), the highest SLP value was obtained (156 W g^−1^) corresponding to an ILP value of 2.6 nHm^2^kg^−1^. This thermal efficiency is superior in comparison with other reported coated magnetite particles (Table 4), including polyethylene glycol (PEG), [61,87] chitosan, [85] polycaprolactone (PCL), [88] dextran, [16] dimercaptosuccinic acid (DMSA) [89], silica, [90] poly-L-lysine, [91] oleic acid, [84] and folic acid [92]. Ghosh et al. [87], through a co-precipitation method, obtained magnetite NP post-synthesis coated with polyethylene glycol (PEG) with a medium size of 10 nm and an ILP value of 0.1 nHm^2^kg^−1^, which is much lower than the value resulted from MF. Dabbagh et al. [61] produced porous magnetite NP and coated them with the same polymer, for hyperthermia and chemotherapy applications, and obtained a smaller ILP value (0.38 nHm^2^kg^−1^). Shaterabadi and collaborators coated magnetite NP with sizes around 18.9 nm with dextran and even after a hydrothermal process, they reached a similar ILP (0.58 nHm^2^kg^−1^) [16]. Moreover, Moorthy et al. [31] synthetized magnetite NP through a solvothermal reaction with a posterior hydrothermal treatment at 200 °C for 12 h and coated them with silica and fucoidan, obtaining NP with sizes around 365 nm and, even with this lower size, a ILP of 1.4 nHm^2^kg^−1^ was obtained. Liu et al. [90] also coated magnetite NP with silica targeting a combined thermotherapy and chemotherapy, achieving NP with 55 ± 10 nm and an ILP of 0.49 nHm^2^kg^−1^.

According to the literature, the application of coatings in situ is more complex, but provide greater stabilization and better magnetic properties [93]. However, the sample MF−IS exhibited a SLP value of 100.26 Wg^−1^ (ILP = 1.7 nHm^2^kg^−1^), lower than the obtained by post-synthesis coating. The reduction of the particle size enhances the surface to volume ratio and the relative contribution of the different aligned magnetic moments at the surface layer leading to a reduction of the Ms value. The reduction of the Ms value may have a negative impact on the thermal efficiency of NP. Furthermore, it is reported in the literature that there is a decrease in the Ms value for sizes below 10 nm [93]. However, according to ILP values reported in the literature even for NP with higher sizes, we achieved NP with better thermal properties and with potential for MHT (see Table 4).

In general, a reduction on SLP value with the fucoidan coating was expected when compared with the bare NP, since a non-magnetic coating could reduce the Ms [93]. However, in this work an increase in the SLP value was observed for the fucoidan coated NP, being the ILP values obtained suitable for MHT for both synthesis methods. This can be explained on light of the better solution dispersion of the NP due to the fucoidan coating as observed through Zeta Potential measurements due to the decrease of interparticle interactions [1]. For instance, Iglesias et al. [94] evaluated the effect of electrostatic and polymeric stabilization of polyethylene oxide (PEO)- coated magnetite NP. It was highlighted a higher hyperthermia performance due to the PEO- coating stabilization effect. Hedayatnasab and co-workers likewise reported this enhanced effect on thermal efficiency, through the coating of magnetite NP (18 ± 2 nm) with PCL, obtaining a final ILP of 1.22 nHm^2^kg^−1^ [88].

### 3.3. In Vitro Cytotoxicity Assay

**Figure 7:**
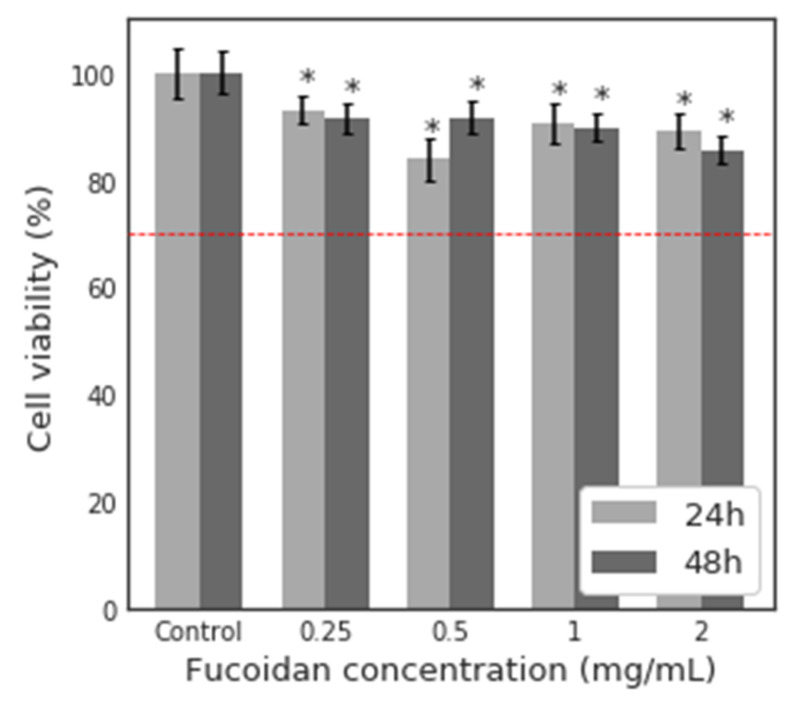
Relative viability (%) of MNT-1 cells, after 24 and 48 h exposure to different concentrations of fucoidan (0.25, 0.5, 1 and 2 mg mL^−1^). Results were expressed as mean ± SD from three independent experiments. Statistical differences between the samples and control are represented by * when *p* < 0.05. The red dotted line at 70% is the toxicity limit, according to ISO 10993-5:2009(E).

MTT assay was performed to evaluate the potential cytotoxicity of fucoidan, pristine magnetite NP (M) and fucoidan-coated magnetite NP after synthesis (MF) and in situ (MF−IS) on highly pigmented human melanoma cells (MNT-1 cell line). Thus, the concentrations of 0.25, 0.5, 1 and 2 mg mL^−1^ were used to evaluate the potential cytotoxicity of fucoidan on MNT-1 cells after 24 and 48 h (Figure 7).

The cytotoxic effects were evaluated according to the standard ISO 10993-5:2009(E), in which a material is considered cytotoxic if cell viability is reduced by more than 30% [95]. It can be seen through Figure 5 that fucoidan is biocompatible for all concentrations and both exposure times with viability higher than 70%, even for the higher concentration (2 mg mL^−1^) and after 48 h of exposure. These results are according with the literature [22], confirming the biocompatibility of this material and their potential use in biomedical applications.

**Figure 8:**
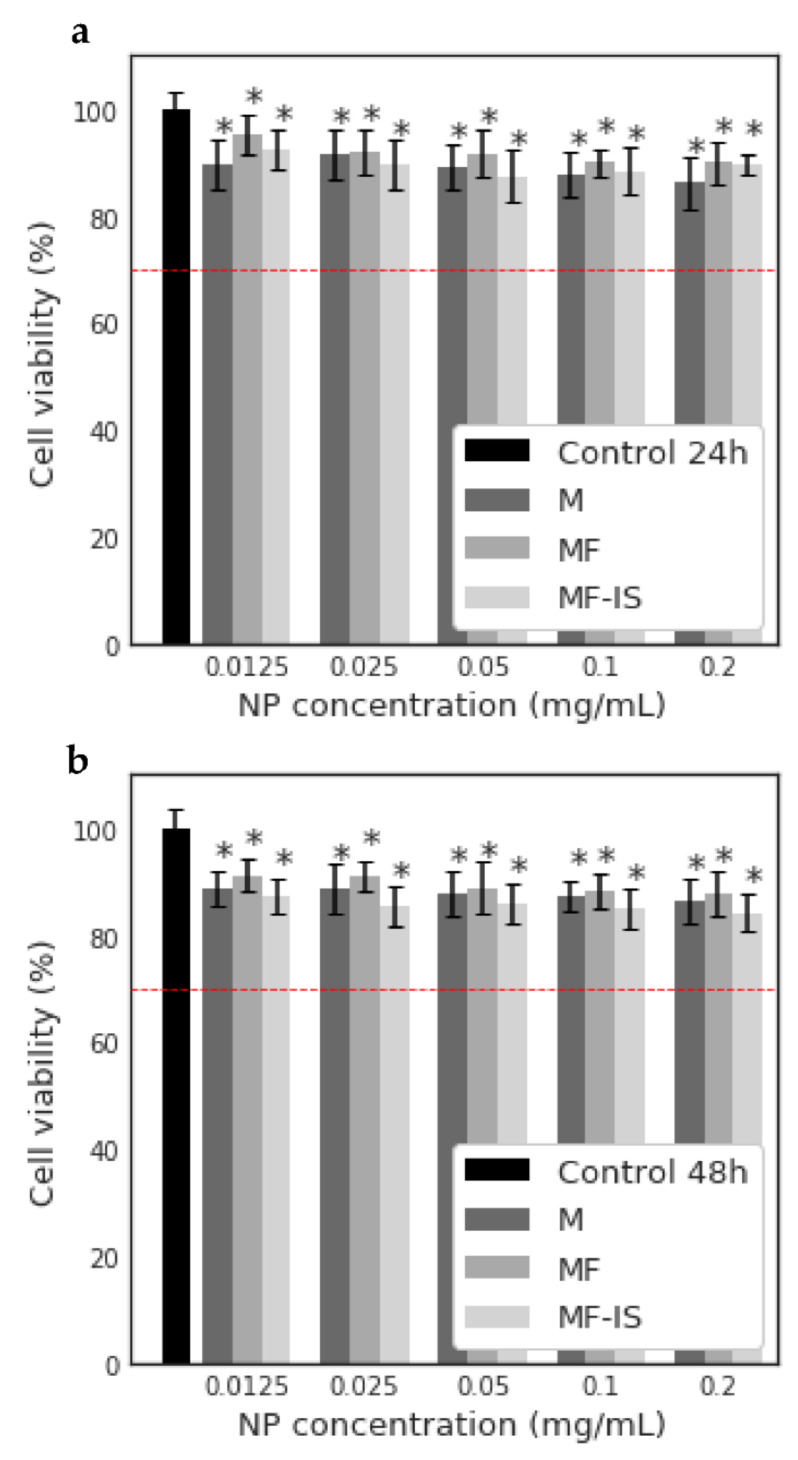
Relative viability (%) of MNT-1 cells after 24 (**a**) and 48 h (**b**) exposure of a range of concentrations (0.0125, 0.025, 0.05, 0.1 and 0.2 mg mL^−1^) of pristine magnetite NP (M), fucoidan-coated magnetite NP post- synthesis (MF) and in situ (MF−IS). Results were expressed as mean ± SD from three independent experiments. Statistical differences between the samples and control are represented by * when *p* < 0.05. The red dotted line at 70% is the toxicity limit, according to ISO 10993-5:2009(E).

The potential cytotoxicity of the synthetized NP (M, MF and MF−IS) was also evaluated on MNT-1 cells for several concentrations (0.0125, 0.025, 0.05, 0.1 and 0.2 mg mL^−1^) after 24 (Figure 8a) and 48 h (Figure 8b). As it can be observed in Figure 8, all the samples were revealed to be biocompatible.

## 4. Conclusions

The NP post-synthesis coated with fucoidan (2 mg mL^−1^) revealed a great thermal efficiency, colloidal stability, and a suitable size, allowing their use for MHT. The NP synthetized and coated simultaneously (in situ preparation) presented a higher agglomeration, which may be associated with their larger surface area when compared to the post-synthesis coated samples, reducing their free surface area. The post-synthesis coated NP showed a 50% higher thermal efficiency comparing to the in situ (SLP 100 Wg^−1^), which allows the tailoring of the NP’ preparation according to the type of MHT treatment required for different cancers.

Both coating methodologies with fucoidan allowed the achievement of SLP values in general higher than the ones reported in the literature for other magnetite NP that were non-coated or coated with other polymers. The coating with fucoidan contributes to a better colloidal stability, as well as avoids NP aggregation, enhancing their thermal efficiency. Furthermore, in vitro assays showed the biocompatibility of these NP.

The fucoidan coated magnetite NP synthetized using a simple and environmentally friendly methodology were shown to be thermally efficient and biocompatible and could be potentially used in MHT.

## Figures and Tables

**Table 1:** Fucoidan monosaccharide composition and sulfate groups content (μg/mg _fucoidan_).

Monosaccharide Composition							Sulfate Content	Total
Fuc	Glc	Man	Xyl	Gal	Rha	Uronic Acids		
418.1	24.8	51.1	17.6	229.6	21.5	75.9	104.5	943.2

**Table 2:** Average crystal sizes measured in TEM image (D_TEM_), average crystallite sizes obtained via X-ray diffraction (D_XRD_) and specific surface area (A_BET_) of magnetite (M), post-synthesis fucoidan-coated magnetite (MF), and in situ fucoidan-coated magnetite (MF−IS) samples.

Sample	D_TEM_ (nm)	D_XRD_ (nm)	A_BET_ (m^2^g^−1^)
M	10.2 ± 3.3	10.8	96.3
MF	11.7 ± 3.1	12.5	91.3
MF−IS	6.4 ± 1.6	8.8	111.8

**Table 3:** Hyperthermia values obtained for all the samples. The SLP measurements were carried out using an AC field amplitude of 14.7 kA m^−1^ at 276 kHz.

Sample	SLP (W g^−1^)	ILP (nHm^2^kg^−1^)
Pristine (M)	30	0.5
Post-synthesis coating (MF)	156	2.6
In situ coating (MF−IS)	100	1.7

**Table 4:** Summarizes reported ILP values for pristine and coated magnetite NP with different coating materials.

Literature Report			
Coating	Medium Size (nm)	ILP (nHm^2^kg^−1^)	Reference
*-*	10-12	0.22	[83]
*-*	8.3	0.23	[84]
*-*	13	0.22	[86]
*-*	21.8	0.42	[85]
Polyethylene glycol (PEG)	10	0.1	[87]
PEG	179	0.38	[61]
Dextran	18.9	0.58	[16]
Chitosan	15.1	0.63	[85]
Dimercaptosuccinic acid (DMSA)	11.4	1.01	[89]
Silica and Fucoidan	365	1.4	[31]
Silica	55	0.49	[90]
Polycaprolactone (PCL)	21	1.22	[88]
Poly-L-lysine	10	1.23	[91]
Oleic acid	10	0.27	[84]
Folic acid	21.1	2.52	[92]

## Data Availability

The data is included in the main text and/or the Supplementary Materials.

## Supplementary Materials

The following are available online at https://www.mdpi.com/article/10.3390/nano11112939/s1, Figure S1: Temperature dependence of the magnetization for MF−IS sample measured at 2 mT, Figure S2: Hysteresis curve at 250 K for MF−IS sample. The inset shows the low field region of the hysteresis curves, Figure S3: Minor hysteresis curves at 250 K for MF−IS sample.
