# Supplementary material for: Coating of Magnetite Nanoparticles with Fucoidan to Enhance Magnetic Hyperthermia Efficiency
Source: Nanomaterials (Basel). 2021 Nov 2;11(11):2939. doi: 10.3390/nano11112939 (PMC8623727; doi:10.3390/nano11112939)
Supplement: Supplementary file 1 [file nanomaterials-11-02939-s001.zip › nanomaterials-1408000-supplementary.pdf]

nanomaterials

MDPI

Coating of Magnetite Nanoparticles with Fucoidan to Enhance Magnetic Hyperthermia Efficiency

Joana Gonçalves [1], Cláudia Nunes [1,*], Liliana Ferreira [2,3], Maria M. Cruz [3], Helena Oliveira [4], Verónica Bastos [4], Álvaro Mayoral [5,6,7], Qing Zhang [7], Paula Ferreira [1,*]

Supplementary Materials:

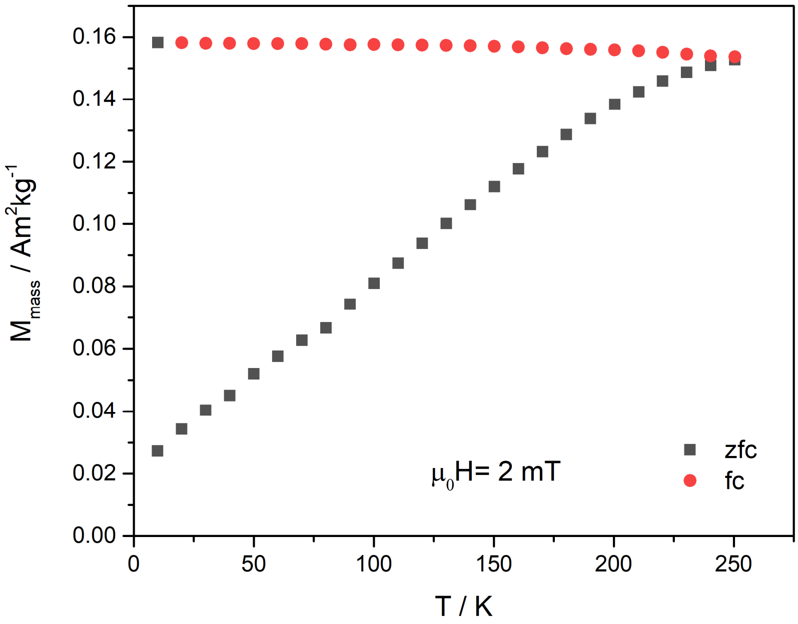

Figure S1: Temperature dependence of the magnetization for MF−IS sample measured at 2 mT.

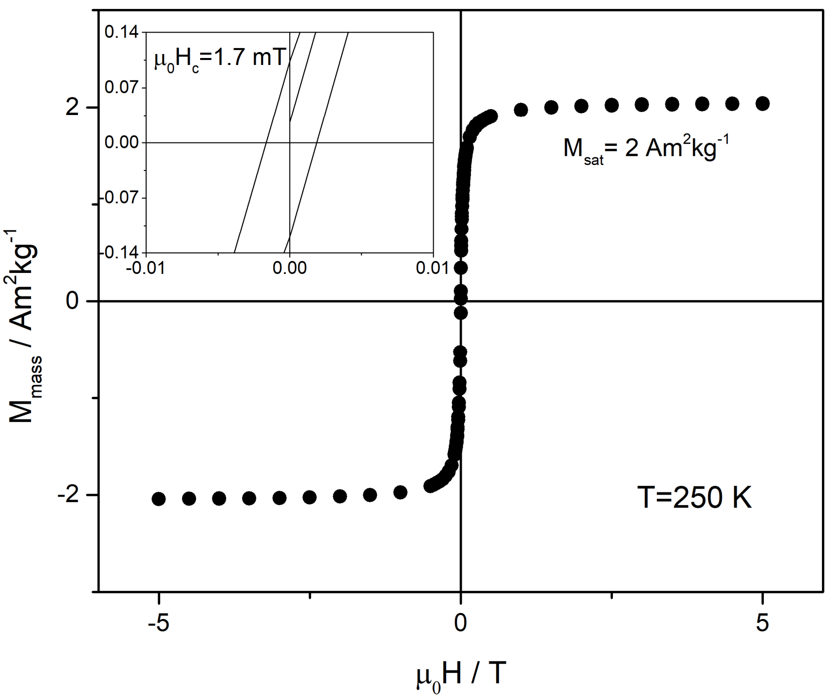

Figure S2: Hysteresis curve at 250 K for MF−IS sample. The inset shows the low field region of the hysteresis curves.

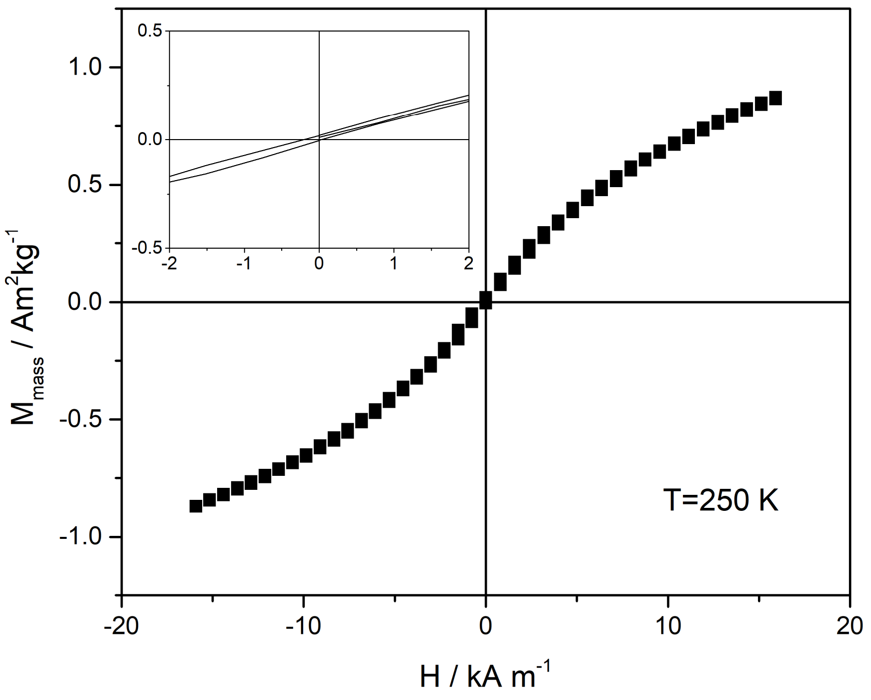

Figure S3: Minor hysteresis curves at 250 K for MF−IS sample.
